# Spatial distribution of soil organic carbon stock in Moso bamboo forests in subtropical China

**DOI:** 10.1038/srep42640

**Published:** 2017-02-14

**Authors:** Xiaolu Tang, Mingpeng Xia, César Pérez-Cruzado, Fengying Guan, Shaohui Fan

**Affiliations:** 1 Key laboratory of Bamboo and Rattan, International Centre for Bamboo and Rattan, Beijing 100102, P.R. China; 2 Departamento de Ingeniería Agroforestal, Universidad de Santiago de Compostela, Lugo 27002, Spain

## Abstract

Moso bamboo (*Phyllostachys heterocycla* (Carr.) Mitford cv. *Pubescens*) is an important timber substitute in China. Site specific stand management requires an accurate estimate of soil organic carbon (SOC) stock for maintaining stand productivity and understanding global carbon cycling. This study compared ordinary kriging (OK) and inverse distance weighting (IDW) approaches to study the spatial distribution of SOC stock within 0–60 cm using 111 soil samples in Moso bamboo forests in subtropical China. Similar spatial patterns but different spatial distribution ranges of SOC stock from OK and IDW highlighted the necessity to apply different approaches to obtain accurate and consistent results of SOC stock distribution. Different spatial patterns of SOC stock suggested the use of different fertilization treatments in Moso bamboo forests across the study area. SOC pool within 0–60 cm was 6.46 and 6.22 Tg for OK and IDW; results which were lower than that of conventional approach (CA, 7.41 Tg). CA is not recommended unless coordinates of the sampling locations are missing and the spatial patterns of SOC stock are not required. OK is recommended for the uneven distribution of sampling locations. Our results can improve methodology selection for investigating spatial distribution of SOC stock in Moso bamboo forests.

Soil is the most important and largest carbon (C) pool in the terrestrial biosphere^1^. However, it is always associated with large variability due to complex composition with variable chemical interactions, human activities, terrain and climate conditions^1^,^2^,^3^. Although considerable effects have been taken into account to accurately estimate SOC pool and its dynamics, the size of the soil organic carbon (SOC) stock derived from different estimation approaches is still inconclusive. For example, global estimates of SOC pool ranges from 504 Pg C (1 Pg = 1 × 10^15^ g) to 3000 Pg C, with a median of 1460.5 Pg C^1^. With the same global database, global C flux from soil ranges from 91 Pg C using a climate driven model^4^ to 98 Pg C using a linear model driven by cell area, leaf area and climate data^5^. This difference amounts to a similar size of fossil fuel combustion^6^ and limits the comparability of measurements. Thus, to overcome these obstacles, it is important to directly compare the approaches for SOC estimation to improve our understanding of global C cycling.

Estimating regional or landscape SOC stock was first undertaken in 1951^7^. The conventional approach (CA) for estimating regional scale SOC stock was undertaken by calculating mean SOC stock in areas using soil type or land use^8^,^9^,^10^. Although this approach is valuable for estimating regional SOC, it may lead to a less reliable estimate of SOC stock due to: (1) the complex interactions between environmental variables; (2) limited representation of assigned average SOC values from few SOC data; (3) the limited representation of spatial distribution and soil forming process; and (4) analytical uncertainties related to determine SOC content, bulk density and soil texture^11^,^12^. These limitations therefore have constrained the use of CA in estimating regional SOC stocks. However, the development of spatial interpolation techniques over the last several decades, such as kriging approaches and inverse distance weighting (IDW), have overcome these problems by: (1) calculating the semivariograms that are expressed as a function of distance between sampled points, and the integrity of spatial continuity in one or multiple directions^13^; (2) employing interpolation techniques (such as spatial interpolation) which calculate unmeasured properties at given places using measured data from neighbouring areas with a weighted mean with limited data points^14^; (3) producing continuous distribution maps of SOC stocks; and (4) examining the accuracy of spatial maps. Therefore, geostatistical analysis has been widely used to predict regional and landscape scale SOC stocks and other soil variables^3^,^15^,^16^.

Bamboo forests are a typical forest type in Southern China (south of the Qinling Mountain-Huai River line), representing an area of 6.16 million hectares^17^; and more than 70% of the bamboo forests consist of Moso bamboo (*Phyllostachys heterocycla* (Carr.) Mitford *cv. Pubescens*)^17^. Moso bamboo forests are noted for fast biomass accumulation; after shoot emergence in spring, this bamboo species can attain full growth (height and diameter) within 35–40 days^11^. After attaining full growth, the diameter and height of the bamboo remain unchanged due to the lack of secondary cambium and it begins to slowly accumulate dry mass^11^. Total C stock in bamboo forests amount to 11% of the total C stock of China’s forest ecosystems^9^, thus bamboo forests play a critical role in regional, national and even global C cycling^18^. In recent decades, due to market prices and sustainable forest development policy, Moso bamboo forests has rapidly expanded^19^. In China, natural forests are protected from felling in order to protect the environment. Consequently, as a large timber consuming county, Moso bamboo has become a major wood substitute and can be harvested every year^20^. Therefore, to maximize the economic benefits for the increasing timber market, intensive management of bamboo forests has been widely applied, such as fertilization and regular understory removal^21^, especially in the main bamboo producing provinces such as Zhejiang and Fujian^22^,^23^,^24^. These management practices have been shown to change soil chemical compositions^25^ and increase soil C mineralization^22^. Thus, to maintain stand productivity, scientific management and estimation of regional scale SOC is necessary because SOC facilitates the growth of soil biota by providing energy from C compounds and nutrients in inorganic forms^26^.

Numerous investigations have been conducted to estimate SOC stock in Moso bamboo forests, for example by Tang, *et al*.^27^ and Xiao, *et al*.^28^. To our knowledge, only one investigation has studied the spatial distribution of SOC stock using ordinary kriging (OK)^29^ and one study has investigated the soil properties in Moso bamboo forests^30^. However, some unresolved questions still remain: (1) is there a consistent result of SOC stock using different geostatistical approaches, such as OK, IDW and CA? and (2) if there is a consistent result in total SOC stock derived from different approaches, is there any difference in the spatial distribution of SOC stock? In addition, site specific maps of SOC stocks for scientific management of Moso bamboo forests are still lacking in our study area. Therefore, this study aims to: (1) study the spatial distribution of SOC stock at four depths (0–20, 20–40, 40–60 and 0–60 cm) in Moso bamboo forests; (2) calculate the SOC pool for the whole study area; (3) compare SOC stock derived from OK, IDW and CA; and (4) compare the spatial distribution of SOC derived from OK and IDW. These objectives will improve our understanding of methodology selection for predicting SOC stock.

## Results

### Descriptive statistics

The summary of descriptive statistics for measured SOC stock is presented in Table 1. Results show that SOC stock showed a decreasing trend with increasing soil depth; for 0–20 cm, SOC stock fell within 20.7–90.0 Mg ha^−1^, and it was 10.5–72.2 Mg ha^−1^ for 20–40 cm, 6.6–80.1 Mg ha^−1^ for 40–60 cm and 52.8–229.7 Mg ha^−1^ for 0–60 cm. Mean SOC stock was 50.9, 42.6, 33.3 and 126.7 Mg ha^−1^ for soil layers 0–20 cm, 20–40, 40–60 cm and 0–60 cm, respectively. The lowest coefficient of variation (CV, 32%) was identified for SOC stock at the 0–60 cm layer while the highest CV (47%) was at the 40–60 cm layer. A CV value of 10% indicates low variability and values ranging from 10–90% indicate a moderate variability; CV values >90% indicate high variability^31^. Therefore, SOC stock in our study area suggested a moderate variability. *p* values for the Shapiro-Wilk test ranged from 0.007 to 0.041, indicating a non-normal distribution of SOC stock for the different soil layers at the 0.05 level of significance. Therefore, before conducting spatial interpolation, a natural log-transformation was undertaken to meet the assumption of normal distribution.

### The relationships between SOC stock and topographic variables

The relationships between topographic variables (elevation, slope and aspect) and SOC stock at the different soil layers were analysed using linear regression. Positive significant relationships between elevation and SOC stock (Table 2) were identified, indicating SOC stock increased with elevation. However, no significant relationship was found between slope, aspect and their interactions, and SOC stock. The interactions between elevation and slope and elevation and aspect led to significant effects on SOC stock, except for SOC stock at 20–40 cm and 40–60 cm layers for the interactions between elevation and slope.

### Spatial autocorrelation and trend surface analysis

Moran’s I value was used to determine the spatial autocorrelation of SOC stocks at different soil layers. Moran’s I value ranged from 0.15 to 0.19 (Table 3), indicating that SOC stocks in all soil layers exhibited a significant positive spatial autocorrelation (all *p* < 0.001). This result concurred with the assumption of OK, that the SOC stocks (regionalized variables) distributed in different locations were spatially correlated. Trend surface analysis indicated that there were significant spatial trends in SOC stocks (Table 3). Second order trend surface explained 27–39% of the variation of SOC stocks. The trend was removed during interpolation of the results.

### Geostatistical analysis

**Figure 1:**
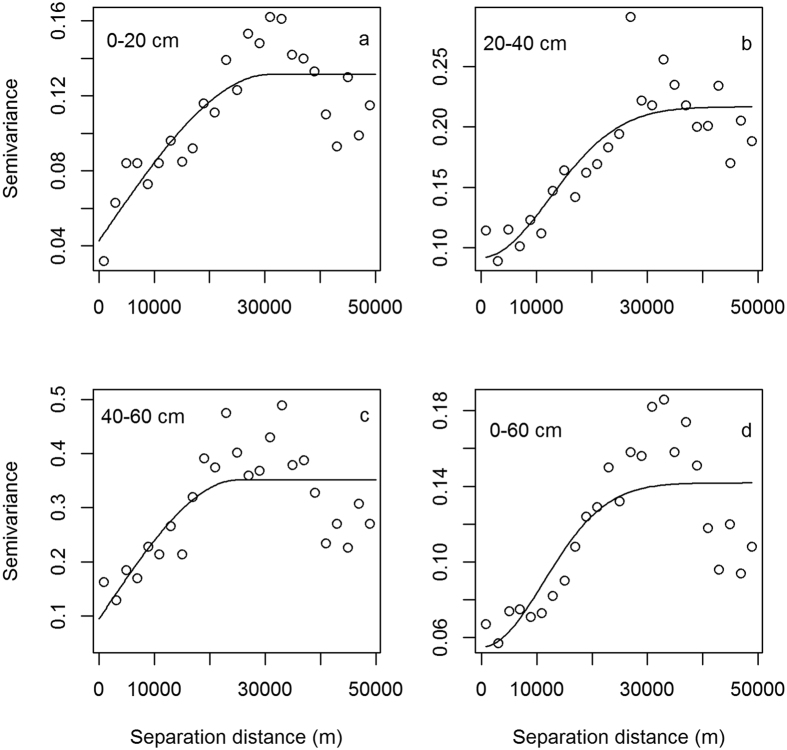
Experimental semivariograms and spatial models for SOC stock at 0–20 cm, 20–40 cm 40–60 cm and 0–60 cm layers.

Experimental semivariograms are presented in Fig. 1 and the parameters are shown in Table 4. Based on the selection criteria of highest determination coefficient and lowest residuals, spherical model can best describe the semivariograms for SOC stock at 0–20 cm and 40–60 cm layers, while Gaussian model best described the results for the 20–40 cm and 0–60 cm layers. The determination coefficient ranged from 0.55 to 0.75 with a residual range of 0.0141–0.109. The semivariogram of SOC stock at the 40–60 cm layer showed a larger Nugget effect than at other soil layers, followed by 20–40 cm, 0–60 cm and 0–20 cm layers. The ratio of Nugget/Sill was lowest for the 40–60 cm (27%) layer and highest for the 20–40 cm (42%) layer. Spatially, the variation ranges decreased from 30,900 m at 0–20 cm to 15,800 m at the 0–60 cm layer.

### Comparison of OK and IDW

**Figure 2:**
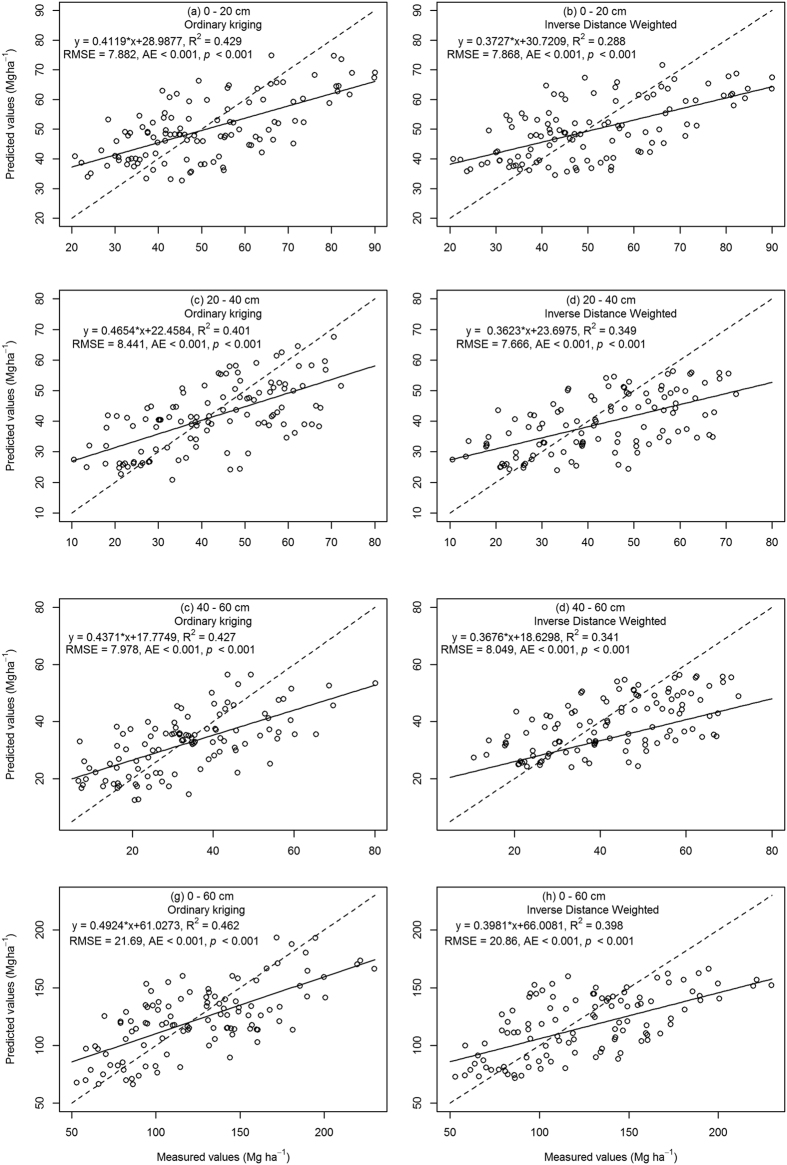
Cross-validation of OK and IDW interpolation for SOC stock at 0–20 cm, 20–40 cm 40–60 cm and 0–60 cm layers (dashed line denotes a 1:1 line).

To check the interpolation performance of OK and IDW, the predicted values were plotted against the measured values (Fig. 2) The linear model intersected 1:1 line for SOC stock. Before the intersection, the linear model (continuous line) overestimated SOC stock, after intersection SOC stock was underestimated. This result was due to the nature of the algorithms used for parameter estimation which aimed to achieve unbiased prediction of the mean values^32^,^33^.

The values of AME, ME, RMSE and pseudo R^2^ are shown in Table 5. The closer the AME, ME and RMSE values are to zero, the better the model performed. ME of SOC stock at different soil layers varied from −0.94 to −6.98 Mg ha^−1^ and determination coefficient of OK and IDW ranged from 0.35 to 0.46, indicating that predicted values derived from OK and IDW slightly underestimated SOC stock, but they were suitable for mapping SOC. Results for OK analysis had a higher determination coefficient and lower AME, ME and RMSE values than IDW for all soil layers, therefore having a better performance. This finding may be attributed to the sampling design and the nature of the algorithm of OK (see the discussion section for more details).

### Spatial prediction of SOC stock

**Figure 3:**
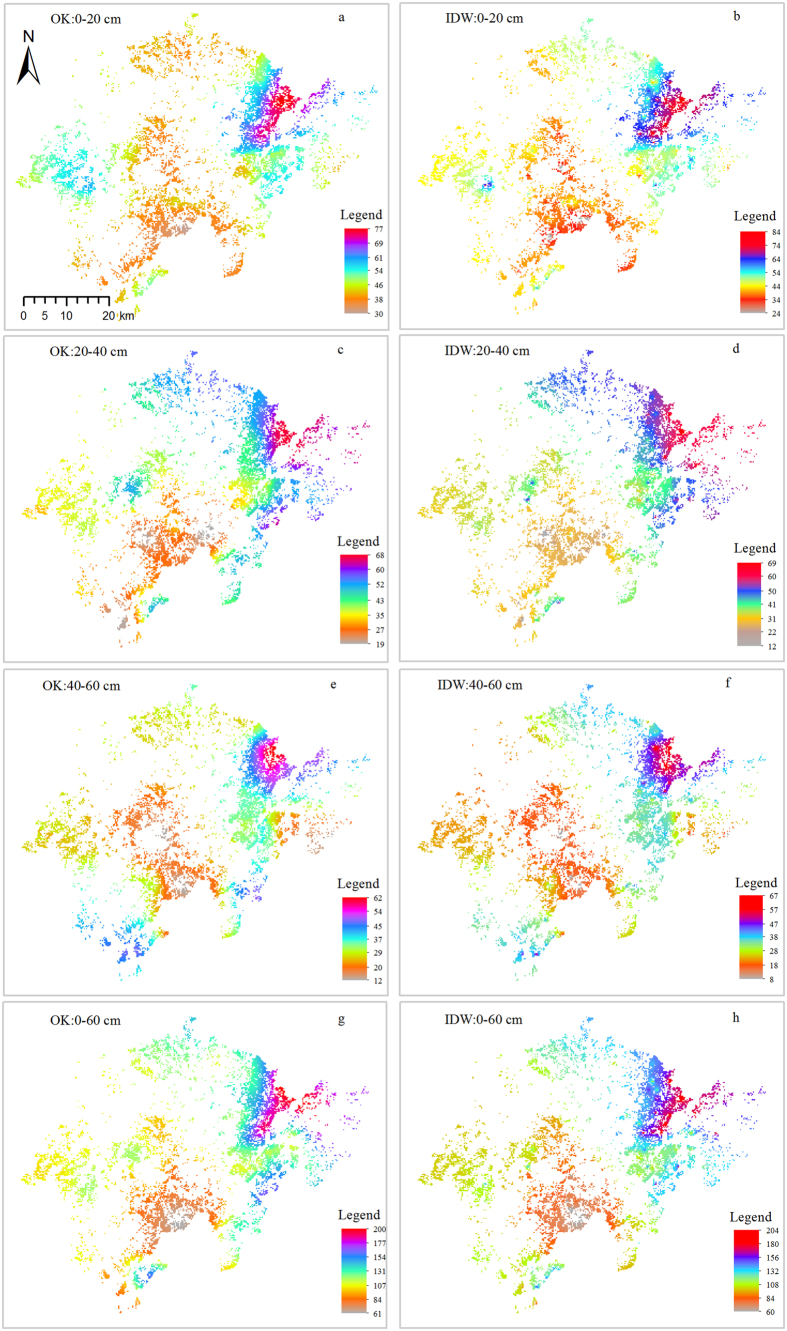
Spatial distribution of SOC stock derived from OK and IDW at 0–20 cm, 20–40 cm 40–60 cm and 0–60 cm layers (non-bamboo areas are excluded). This figure was generated using ArcMap 10.2 (http://www.esri.com/).

To further compare the differences of OK and IDW for the spatial interpolation of SOC stock at different soil layers, spatial distribution maps were produced using both OK and IDW (Fig. 3); results of which showed a strong spatial variability of SOC stock across the whole study area. Generally, SOC stocks were highest in the northeast of the study area for all soil layers and lowest in the central areas, correlating to city centre locations. For 0–20 cm, 20–40 cm, 40–60 cm and 0–60 cm layers, SOC stock derived from OK fell by 30–77, 19–68, 12–62 and 61–200 Mg ha^−1^, respectively. These ranges were lower than those derived from IDW. The distribution patterns for OK and IDW were generally similar, however the absolute values of SOC differed. For example, SOC stock for the 20–40 cm layer in the southern part of the study area varied from 45–60 Mg ha^−1^ for OK (Fig. 3c) and 35–45 Mg ha^−1^ for IDW (Fig. 3d).

### Total SOC stock prediction

The SOC pool from the different soil layers are shown in Table 6. Although different ranges of SOC stock derived from OK and IDW were observed amongst the different soil layers (see above description), SOC pool derived from OK and IDW were comparable (1.67–2.59 Tg for OK and 1.61–2.58 Tg for IDW). However, SOC stock estimated from CA was higher than that of OK and IDW for the different soil layers. Total SOC pool of Moso bamboo forests within the study area was 6.46 Tg for OK, 6.22 for Tg and 7.41 Tg for CA.

## Discussion

Soil is the largest C pool and accurate estimation of SOC is therefore necessary to assess C sequestration or emission potential caused by global environmental changes^1^. SOC stock in the top 60 cm of the Moso bamboo forests was 126.7 Mg ha^−1^, falling within the SOC stock range of other major forests (16–572 Mg ha^−1^)^34^, and being greater than the average SOC stock of main forest types (78 Mg ha^−1^) in China^34^. This result indicates that the Moso bamboo forest soil is a larger carbon pool compared to the average soil results of other forest types. In south China, driven by fast and high economic benefits due to annual harvests, the area of Moso bamboo is increasing by the rate of 3% per year^35^. The increase of Moso bamboo forest area indicates an increase of SOC stock in Moso bamboo forests under the intensive management. In addition, the carbon stock of (including above- and below-ground) Moso bamboo is dynamically balanced between annual harvest and growth of new bamboos, and one sixth of aboveground biomass is harvested for timber every year^36^, thus leading to a higher ecosystem production than the fast-growing Chinese fir plantations^28^. Our results suggest that Moso bamboo forest soils play an important role in alleviating future climate change. In contrast, deforestation of Moso bamboo forests could lead to significant losses of carbon to the atmosphere, thus causing a negative feedback to climate change.

SOC stock results were also higher than those from Moso bamboo forests in Hubei Province (65–99 Mg ha^−1^)^27^, Zhejiang Province (94 Mg ha^−1^)^29^ and Jiangxi Province (111 Mg ha^−1^)^28^. The higher SOC results of our investigation are mainly attributed to fertilization treatment in the majority of Moso bamboo forests in our study area to improve stand production and bamboo shoots; the other three comparison bamboo forests did not receive fertilization treatments^27^,^28^,^29^. SOC stock in this study was also higher than the average SOC stock of plantations (104 Mg ha^−1^), and it was comparable to natural forests across China (129 Mg ha^−1^)^37^. This indicates that Moso bamboo forests act as an important role in global C cycling with storing more C in soil.

SOC stock results decreased as soil depth increased, a finding which is in general accordance with the majority of previous investigations: SOC in Moso bamboo forests showed a diminishing trend^38^,^39^. However, some investigations also reported a positive trend of SOC stock with increasing soil depth^40^; a finding which we attribute to an unusual trend due to mixing effects of tillage^40^.

Many approaches have been developed in the framework of geostatistical analysis techniques for spatial interpolation, such as OK, co-kriging and universe kriging. As the OK technique normally performs better and is easy to apply, it has been widely used in spatial interpolation of SOC stock^3^,^29^ and soil physical properties^30^. To describe the spatial variability of soil properties, semivariograms are fitted and used^3^,^13^,^29^.

Nugget values present undetectable experimental errors, field variation within the minimum sampling space and inherent variability^41^. Nugget values in this study were small and varied from 0.0427 to 0.0947, indicating a positive nugget effect, a weak field or random variability^41^. The nugget-to-sill ratio represents a spatial dependence. The ratios <25%, 25–75% and >75% suggest a strong, moderate and weak spatial dependence^42^. A strong spatial dependence of soil properties is attributed to soil intrinsic properties, such as soil parent materials, soil texture, topography and vegetation^29^,^41^. On the other hand, a weak spatial dependence of soil properties indicates that the spatial variability is mainly regulated by extrinsic variations, such as soil fertilization and cultivation practices^41^,^43^; moderate spatial dependence is controlled by both intrinsic and extrinsic factors. In this study, the nugget-to-sill ratios ranged from 27% to 42%, demonstrating a moderate spatial dependence of SOC stock for the different soil layers which was controlled by both intrinsic and extrinsic factors. This was evidenced by local, intensive stand management practices, such as tillage and fertilization, widely used in the study area to improve the stand productivity and bamboo shoot output^23^,^44^, and the complex local topography. A previous study also showed that intensive management led to a decrease of soil organic matter content in Moso bamboo forests^19^. This study also detected a significant positive relationship between SOC stock and topographic variables, notably elevation (Table 2). This result also supported findings from previous studies which showed that SOC stock increased with elevation due to a reduction in human disturbance and slower decomposition rates of soil organic matter^19^,^45^. In areas with high rates of disturbance, for example city centres and surround areas, SOC stock was notably lower than areas without disturbance (Fig. 3). Soil temperature, a key determinant of soil organic matter decomposition, decreases with increased elevation which therefore decreases the loss of soil carbon to the atmosphere^45^.

Ranges (A_0_) indicate different influence zones of environmental factors at different scales, beyond the range, the measured data are not spatially dependent^41^,^46^, which means that the sampling points cannot be applied for spatial interpolation. This suggests that A_0_ can therefore be an effective criterion for evaluating sampling design and SOC stock mapping. The lowest A_0_ (15,800 m) was found at the 0–60 cm layer and the largest A_0_ (30,900 m) was found for the 0–20 cm layer. These ranges were larger than the sampling intervals (Fig. 1), which suggested that the sampling strategy in this study was appropriate for studying the spatial pattern of SOC stock. However, A_0_ differed from soil layers, indicating that the influence zones of SOC stock were not uniform and were depth dependent. This result was mainly associated with intensive human disturbance during timber harvest, digging bamboo shoots and applying fertilizers. These processes lasted for more than six months per year and mainly occurred within the 0–40 cm soil layers where bamboo roots and shoots are widely distributed^27^.

**Figure 4:**
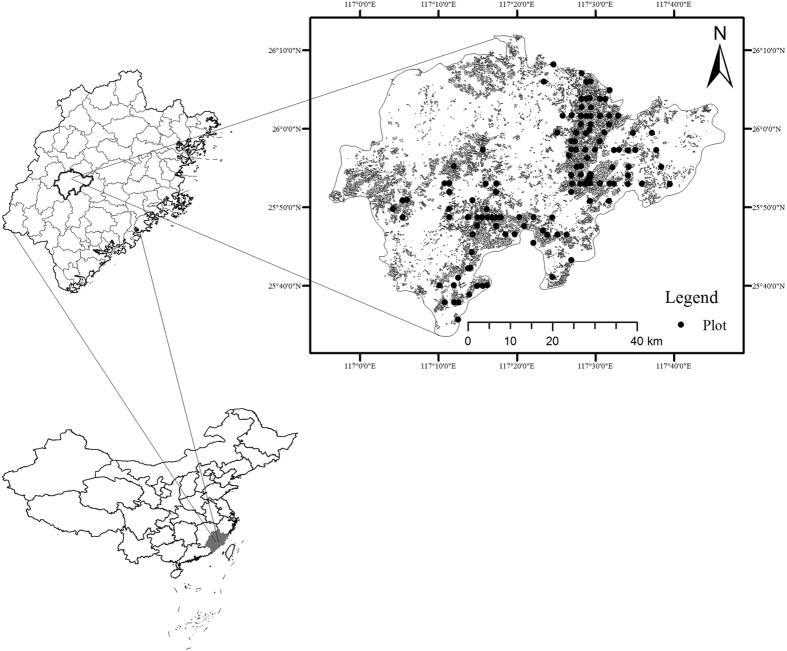
Study area, the distribution of bamboo forests (grey polygons) and sampling locations (solid black circle). This figure was generated using ArcMap 10.2 (http://www.esri.com/).

ME values in our analysis were negative, indicating both OK and IDW underestimated SOC stock. OK analysis had higher R^2^ values and lower AME, ME and RMSE results suggesting this analysis had a more suitable fit than IDW, a finding which is supported by previous studies^14^,^32^,^47^. The R^2^ values, although being relatively low (0.35 to 0.46) were consistent with previous reports on the spatial interpolation of soil properties^29^,^48^. This problem is associated with the dataset that soil samples were not collected using a probabilistic sampling design^48^. In our study, due to uneven distribution of bamboo forest, the majority of sampling plots were located in the southern and eastern areas of Yong’an City (Fig. 4). It is also recommended that probabilistic-based design and depth function should be applied to further study of the vertical distribution of SOC stocks since the fertilization treatment could considerably affect the nutrient supply and availability, especially for the top layers, where most of roots are distributed. Another possible explanation for low R^2^ values might be the strong local variation of SOC stock due to the variability in the environmental conditions in the study area. However, the low correlation between the predicted and measured data indicated that a better methodology such as Artificial Neural Network or Random Forest may improve the accuracy of spatial interpolation of SOC stock, including both intrinsic and extrinsic factors.

Generally, OK and IDW produced similar results for the spatial pattern of SOC stock, together with comparable total SOC stock derived from OK and IDW for different soil layers, demonstrating the suitability of OK and IDW for spatial interpolation of SOC stock. However, a larger range was produced by IDW compared to OK, indicating the necessity of using different approaches to study the spatial SOC stock, particularly areas with a complex topography. This issue is related to the different algorithms and computational efficiencies for the spatial interpolation of OK and IDW, thus selection of the appropriate approach is important to improve the interpolation accuracy and efficiency. Theoretically, OK can provide the best linear unbiased estimations and information on the spatial patterns of estimation errors^32^. However, it is important to note that the assumption of stationarity may be not appropriate in practice^49^. The IDW method involves a simple and quick calculation and does not require assumptions about the data. However, IDW does not have the statistical advantages compared to OK (Table 4). The IDW formula has the effect of giving data points close to the interpolation point relatively large weights, while points further away from the interpolation point exert little effect^50^. The higher the weighting used, the more influence the point close to the estimation value is given. Therefore, as a result of the irregular distribution of the sampling locations, combined with better statistical performances, OK is recommended as a more suitable approach for similar studies in the future.

Although OK and IDW generated a similar result of SOC pool of Moso bamboo forests for the study area, SOC pool derived from CA was higher than that of OK and IDW, demonstrating the importance of selecting the appropriate approach to estimate SOC stock. Although CA is a simple approach once the mean SOC stock per unit and area of the study site are known, CA is unable to provide information about the continuous mapping of SOC stock and therefore cannot test the accuracy of spatial distribution of SOC stocks. CA can only provide limited information for optimizing stand management to improve stand productivity, thus making CA of limited use in studying the spatial pattern of SOC stock. Therefore, CA is only recommended to be used when the coordinates of the sampling locations are missing and the spatial patterns of SOC stock are not required; different geostatistical approaches are recommended to be used to obtain accurate and consistent spatial patterns of SOC stock and regional SOC pools.

Compared to other studies on SOC stock in Moso bamboo forests, such as Zhang, *et al*.^45^ and Fu, *et al*.^29^, this study is the first to attempt to (1) compare different spatial interpolation approaches; and (2) compare geostatistical approaches and CA for regional SOC stock estimates. These results can improve the methodology selection of studying spatial distribution of SOC stocks. In addition, scientific management of Moso bamboo forests requires site specific maps of SOC stock to improve stand productivity. Regarding stand management, this study further proposed that rather than using a consistent treatment of fertilizers across the whole study area, different distribution patterns of SOC stocks indicated different fertilizer treatments should be conducted in different sites since SOC is an important indicator for soil fertility. For example, organic fertilizer treatment could be applied in the centre areas of the southern study regions (Fig. 3). Together with organic fertilizers, the addition of other nutrients, such as nitrogen, phosphorus and potassium, should be added since, nitrogen and phosphorus are the most important limited nutrients in Moso bamboo forests in south China^51^.

Regarding to uncertainties, analysis of random soil cores for the presence of stones and rocks suggested low contents (<5%), thus we did not correct for gravel content. This could be a source of uncertainty for regional SOC stock estimates, especially in nutrient poor soils. However, data from poor soils is sporadic as the stands were fertilized and managed every year. The estimates of SOC stock from OK and IDW differed by 13% and 16%, respectively, compared to CA. This difference is due to the lack of the relative area weighted mean of CA. Although model efficiency of different models fitted for semivariograms ranged from 55% to 75% (Table 5), R^2^ of the model values and predicted values varied from 0.35 to 0.46. This could be an important uncertainty of regional estimates of SOC stock. However, despite the uncertainty in model efficiency, both geostatistical interpolation (OK) and deterministic (IDW) approaches compared and produced similar estimates of regional SOC stock (3.8% difference, Table 6). This result highlighted that the estimates of total SOC stock were accurate.

## Conclusions

In this study, OK and IDW were applied to study the spatial interpolation of SOC stock at 0–20 cm, 20–40 cm and 40–60 cm using the measured data from 111 plots in Moso bamboo forests in Yong’an City, subtropical China. OK, IDW and CA were applied to estimate the regional SOC pool. These results can facilitate the accurate estimation of spatial distribution of SOC stock and regional SOC pool.

Spherical and exponential models were selected to describe the spatial pattern of SOC stock. A moderate spatial dependence of SOC stock was observed, indicating that SOC stock was controlled by both intrinsic factors (e.g. soil parent materials and soil texture) and extrinsic factors (e.g. application of fertilizers and tillage treatment).

OK and IDW produced similar spatial patterns of SOC stock, together with comparable SOC pool, indicating the suitability of both approaches in studying the spatial interpolation of SOC stock. However, OK produced a smaller distribution range of SOC stock compared to IDW, highlighting that it is essential to apply different approaches to obtain accurate and consistent results of SOC stock distribution. SOC pool derived from CA was higher than that from OK and IDW, thus CA is not recommended unless coordinates of the sampling locations are missing and the spatial patterns of SOC stock are not required.

## Materials and Methods

### Study area

The study area was located in the Yong’an City, Fujian Province, China (117°56′–117°47′E, 25°33′–26°12′N, Fig. 4). The area is characterized by a subtropical southeast monsoon climate with an average annual temperature of 19.3 °C (ranging from −11 °C to 40 °C) and precipitation of 1600 mm^44^,^52^. Elevation in the study area spans 580 m to 1605 m above sea level^44^,^52^. The accumulated temperature of ≥10 °C is 4,520–5,800 °C, lasting for 225–250 days and relative humidity is about 80%^44^. The forest cover is 82% with 5.85 × 10^4^ ha of Moso bamboo forests^52^. Moso bamboo forests are mainly distributed below 800 m, most of which are pure stands and are seldom mixed with *Keteleeria cyclolepis, Cunninghamia lanceolata, Myrica rubra, Choerospondias axillaris, Liriodendron chinense, Schima Superba*, etc. To improve the stand output and increase income, fertilizers have been widely applied to most of the Moso bamboo forests.

### Soil sampling

Soil samples were collected from the sub-compartment of the forest resource management of Fujian province, China, an area which was established by the local Forest Bureau for soil mapping (Fig. 4). In the targeted sub-compartment, a cluster of three circular plots with a size of 33.3 m^2^ were established, and 138 clusters were created in total. However, due to soil sample damage during transportation, soil samples from 111 plots were used for spatial interpolation of SOC stock. In each plot centre, soil samples from three layers (0–20 cm, 20–40 cm and 40–60 cm) were collected. Soil samples were air-dried at room temperature in the laboratory and prepared for sieving through 2-mm and 0.15-mm meshes for SOC content analysis. Identifiable plant residues and root materials were removed during sieving. As the majority of bamboo roots were distributed within the top 40 cm^27^, soil samples to a depth of 60 cm was deemed suitable to meet the research aims of this investigation. To determine bulk density, a cutting ring approach was used in the soil cores^53^. During fertilizer treatment in the plots, identical stones and rocks were removed from the Moso bamboo forests. This resulted in few stones and rocks being found in the cores, therefore correction for gravel content was not undertaken. Information about sample elevation, coordinates, soil depth, soil type, nitrogen content, phosphorus content and bamboo diameters were recorded and determined according to State Forestry Adiministration^53^.

SOC content was analysed using the K_2_Cr_2_O_7_-H_2_SO_4_ wet oxidation method^53^. Specifically, 0.1–0.5 gram of air-dried soil was passed through a 0.15-mm sieve and digested with 5 mL 0.8 mol L^−1^ K_2_Cr_2_O_7_ and 5 ml concentrated H_2_SO_4_ (1.84 g mL^−1^) for 5 min at 170–180 °C. The digested solutions were then titrated using standardized 0.2 mol L^−1^ FeSO_4_ solution mixed with 15 ml concentrated H_2_SO_4_ per liter to prevent oxidization^53^. SOC stock was calculated as^39^,^54^:


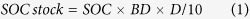


where, SOC is the soil organic C concentration (g kg^−1^); BD is bulk density (g cm^−3^); and D is the depth of the soil layer (cm).

### Extraction of topographic variables from a Digital Elevation Model (DEM)

A DEM with a resolution of 90 m was obtained from Geospatial Data Cloud (http://www.gscloud.cn/). Mean values of aspect, elevation and slope were extracted for each sample plot in ArcGIS 10.2 (http://www.esri.com/). Further details of the calculation of aspect, elevation and slope are described by Pierce, *et al*.^55^.

### Statistical and geostatistical analyses

Traditional statistical analysis, such as mean, standard deviation and coefficient of variation, were calculated to describe the original data. The relationships between topographic variables (elevation, slope and aspect) were analysed using linear regression. Before starting geostatistical analysis, raw data was initially tested for normality using the Shapiro-Wilk test in R^56^. Instances where the data did not meet the assumption of normal distribution, the raw data was log-transformed and then transformed back using weighting mean in GS + 10.0 (www.gammadesign.com). In this study, OK and IDW were applied to estimate the spatial distribution of SOC stock.

### Spatial autocorrelation and trend surface analyses

Moran’s index (I), a common indicator of spatial autocorrelation^57^, provides negative or positive values. A Moran’s I value greater than 0 means a positive spatial autocorrelation (high values or low values cluster together), while values less than 0 indicate a negative spatial autocorrelation (a checkerboard pattern); 0 values indicate perfect spatial randomness^41^. Trend surface analysis is the most widely used surface-fitting procedure. The target soil variables are expressed by a polynomial model of geographic coordinates, and the coefficients of the polynomial model are modelled by the method of least squares^58^. In this study, second degree of polynomial surface was used because the increase of degree did not result in a significant increase of determination coefficient and F ratio.

### Ordinary Kriging (OK)

Kriging is based on the theory of regionalized variables which are spatially distributed and autocorrelated^59^. The spatial autocorrelation can be indicated by Moran’s I (see above). Spherical, exponential and Gaussian models are commonly used to calculate experimental semivariograms using the observed data^49^. The semivariograms are expressed as a function of distance between sampled points and calculate the integrity of spatial continuity in one or multiple directions using the following expression^13^:


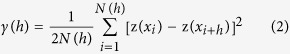


where, *i*, z(*x*_*i*_) and z(*x*_*i+h*_) are values of z at locations *x*_*i*_ and *x*_*i+h*_, respectively; *h* is the lag and *N(h)* is the number of pairs of sample points separated by *h*. In this study, spherical, exponential, linear and Gaussian models were used to describe the semivariograms of SOC stock at 0–20 cm, 20–40 cm and 40–60 cm layers. The models with highest coefficient of determination and the smallest of residuals were chosen. These models where then applied to analyse spatial structure and to provide the input parameters for interpolation.

There are three major parameters derived from the fitted models to identify the spatial structure of SOC stock for a given scale. The parameters are nugget (C_0_), the sill (C + C_0_) and the range (A_0_).

The sill (C + C_0_) represents total variation, and the ratio of nugget and sill is considered as a criterion to classify spatial dependence^3^; A_0_ represents the separation distance, beyond which the measured data are not spatially dependent^46^. More details about the semivariograms and kriging can be found in Goovaerts^49^. The most likely value *R(x)*, which was expected to be encountered in a particular grid cell when using *m* neighbouring observations, was defined as:


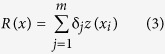


### Inverse distance weighting (IDW)

Similarly, IDW is another important approach for spatial interpolation which assumes that each point influences the resulting surface to a finite distance^60^. IDW calculated an unsampled point as a weighting average of a known data point within local surroundings. The formula can be expressed by Eq. (4) 50. In this study, data points of 16 without a fixed radius and the weight power of one were used; the weight power of one was found to perform better than the weight powers of two, three and four if the skewness is below one^32^,^61^,^62^:


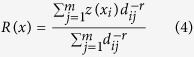


where, *r* is the weight and *d*_*ij*_ is the distance, which is the distance between the estimation point and the measured point.

### Data validation

The prediction accuracy of SOC stock was evaluated using the leave-one-out cross-validation techniques^3^,^33^,^47^. In the determination of errors, one point was omitted and this value was estimated by the remaining values. Afterwards, the estimated value was compared with the real value in the situation of omitted point^3^. This process was repeated for all the observations. Four commonly used indices, i.e., absolute mean error (AME), mean error (ME), root mean square error (RMSE) and model efficiency (R^2^), were used to compare the interpolation accuracy for OK and IDW. These indices were calculated as follows:


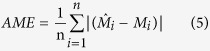



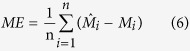



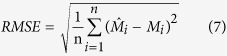



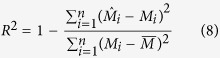


where, 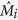
*, M*_*i*_ and 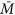 are predicted values, observed values and the mean value of the observations, respectively.

## Additional Information

**How to cite this article**: Tang, X. *et al*. Spatial distribution of soil organic carbon stock in Moso bamboo forests in subtropical China. *Sci. Rep.* **7**, 42640; doi: 10.1038/srep42640 (2017).

**Publisher's note:** Springer Nature remains neutral with regard to jurisdictional claims in published maps and institutional affiliations.

## Figures and Tables

**Table 1:** Statistical description of soil organic carbon stocks for different soil layers (Mg ha^−1^).

Layers	Mean	Minimum	Maximum	Median	SD	CV (%)	1^st^ Qu	3^nd^ Qu	Skewness	Kurtosis	*p* of S-W test
0–20 cm	50.9	20.7	90.0	47.4	16.5	32.42	38.7	62.8	0.45	−0.58	0.009
20–40 cm	42.6	10.5	72.2	43.1	15.4	36.15	29.6	56.0	−0.05	−1.05	0.028
40–60 cm	33.3	6.6	80.1	32.1	15.7	47.15	21.1	42.9	0.44	−0.23	0.007
0–60 cm	126.7	52.8	229.7	119.9	40.6	32.04	93.7	150.6	0.36	−0.58	0.041

SD = standard deviation; CV = coefficient of variation; 1^st^ Qu = 25% quartile; 3^rd^ = 75% quartile; S-W test = Shapiro –Wilk test.

**Table 2:** Coefficients of the relationships between elevation (ele), slope (slo), aspect (asp) and soil organic carbon stocks in different soil layers.

Soil layers	Ele	Slo	Asp	Ele:Slo	Ele:Asp	Slo:Asp
0–20 cm	0.019^**^	3.38 × 10^−4^	−0.006	4.6 × 10^−4*^	4.0 × 10^−5*^	−1.7 × 10^−4^
20–40 cm	0.014^**^	−0.040	0.008	3.9 × 10^−4^	3.76 × 10^−5*^	4.4 × 10^−4^
40–60 cm	0.018^**^	−0.149	0.029	2.91 × 10^−4^	5.8 × 10^−5**^	4.02 × 10^−4^
0–60 cm	0.050^**^	−0.170	0.029	1.23 × 10^−3*^	1.28 × 10^−4**^	8.01 × 10^−4^

* and ** indicates significant difference at level of 0.05 and 0.01, respectively.

**Table 3:** Moran’s I analysis and second-order trend surface analysis of polynomial surface for soil organic carbon stocks in different soil layers.

Soil layers	Moran’s I		Trend surface	
	Estimates	*p*	R^2^	*p*
0–20 cm	0.1616	<0.001	0.2678	<0.001
20–40 cm	0.1635	<0.001	0.3931	<0.001
40–60 cm	0.1528	<0.001	0.2654	<0.001
0–60 cm	0.1928	<0.001	0.3486	<0.001

**Table 4:** Models and their parameters fitted for semivariograms of SOC stocks for different soil layers.

Soil layer	Model	Nugget (C_0_)	Sill (C_0_ + C)	Nugget/Sill (%)	Range (A_0_)	Model efficiency (R^2^)	Residuals
0–20 cm	Spherical	0.0427	0.1314	32.49%	30900	0.694	0.0078
20–40 cm	Gaussian	0.0918	0.2166	42.38%	17500	0.746	0.0164
40–60 cm	Spherical	0.0947	0.3514	26.95%	25200	0.550	0.1090
0–60 cm	Gaussian	0.0549	0.1418	38.72%	15800	0.616	0.0141

**Table 5:** Cross-validation indices for ordinary kriging (OK) and inverse distance weighting (IDW) methods.

Layers	Methods	AME	ME	RMSE	Pseudo R^2^
0–20 cm	OK	10.6770	−0.9439	12.4573	0.429
	IDW	10.9094	−1.2024	12.9752	0.381
20–40 cm	OK	9.5465	−1.1636	11.9914	0.401
	IDW	10.3646	−3.4654	12.8668	0.349
40–60 cm	OK	9.5449	−0.9499	11.8575	0.427
	IDW	10.0389	−2.4074	12.9184	0.341
0–60 cm	OK	24.5449	−2.3665	29.8196	0.462
	IDW	26.3927	−6.9758	32.1667	0.398

AME = absolute mean error; ME = mean error; RMSE = root mean square error; Pseudo R^2^ = pseudo determination coefficient.

**Table 6:** SOC pool for different soil layers (1 Tg = 1 × 10^12^ g).

Soil layers	Approaches	SOC stocks (Tg)
0–20 cm	OK	2.59
	IDW	2.58
	CA	2.98
20–40 cm	OK	2.28
	IDW	2.15
	CA	2.49
40–60 cm	OK	1.67
	IDW	1.61
	CA	1.95
0–60 cm	OK	6.46
	IDW	6.22
	CA	7.41
